# Reply to “Comment on ‘Efficacy of electroacupuncture in improving postoperative ileus in patients receiving colorectal surgery: a systematic review and meta-analysis’”

**DOI:** 10.1097/JS9.0000000000001423

**Published:** 2024-04-09

**Authors:** Kuo-Chuan Hung, Chia-Li Kao, Hsiao-Tien Chen, I-Wen Chen

**Affiliations:** Departments of a Anesthesiology; b Chinese Medicine, Chi Mei Medical Center, Tainan; c Department of Anesthesiology, E-Da Hospital, I-Shou University, Kaohsiung City; d Department of Anesthesiology, Chi Mei Medical Center, Liouying, Tainan city, Taiwan

*Dear Editor*,

We recently conducted a systematic review and meta-analysis to evaluate the efficacy and safety of electroacupuncture in improving postoperative ileus after colorectal surgery^1^. Our study included 16 randomized controlled trials (RCTs) with a total of 1562 patients. Pooled results from these articles revealed that patients who received electroacupuncture had shorter time to first flatus, first defecation, and bowel sound recovery than those who received standard care. Liang *et al*.^2^ recently reported several concerns regarding our previous work. We would like to thank Liang *et al*.^2^ for their interest in our article^1^ and thoughtful comments. We wish to address these concerns and provide further clarifications.

First, Liang *et al*.^2^ pointed out that one RCT^3^, previously cited in an earlier meta-analysis^4^, was omitted from our current analysis. This exclusion could potentially have affected the robustness of our results. However, it should be noted that the article^3^ mentioned by Zhao *et al*.^4^ could not be identified in major databases (e.g. PubMed) or Google Scholar, which likely led to its omission from our initial search. Our evaluation of Zhao *et al*.^4^‘s work revealed that they categorized this study^3^ as having a high risk of bias. Additionally, the length of only two pages in the article^3^ raises concerns regarding its comprehensiveness and suitability for inclusion in our meta-analysis. Consequently, the absence of this article^3^ is not expected to have a significant impact on the overall conclusions of our findings.

Second, Liang *et al*.^2^ highlighted the inclusion of a study^5^ in the Results section that was not relevant to the subject of this research, raising concerns about the accuracy of our meta-analysis. We would like to clarify that this article^5^ in question was incorrectly cited in one sentence in the Results section^1^. We apologize for the confusion this may have caused. However, as evident from Table 1 of our original meta-analysis^1^, which lists the studies included in the final analysis, this article^5^ in question was not included in our analysis.

Third, we appreciate the suggestion by Liang *et al*.^2^ of employing more objective tests to identify potential publication bias. To address this concern, we reanalyzed our data using Egger’s test. Comprehensive Meta-Analysis software (Version 4, Biostat) was used for the analysis. As shown in Fig. 1, there was no evidence of publication bias for the primary outcome (i.e. time to first flatus) based on the result of Egger’s test (*P*=0.41).

**Figure 1:**
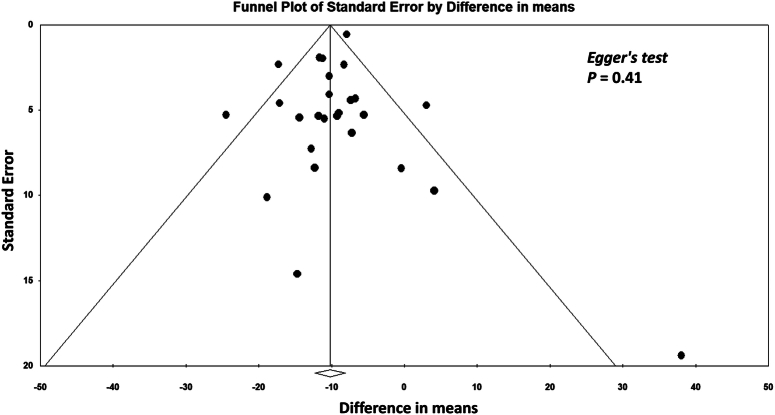
Funnel plot assessing publication bias in the analysis of studies on the effect of EA on time to first flatus. Each point represents an individual study and is plotted according to the standard error and difference in means. The vertical line indicates the pooled estimate of the difference in means, whereas the diagonal lines represent the expected distribution of studies in the absence of publication bias. Egger’s test (*P* = 0.41) suggests no significant evidence of publication bias in this meta-analysis. EA, electroacupuncture.

We would like to thank Liang and colleagues^2^ once again for their valuable input. We hope that our responses have adequately addressed their concerns and have provided the necessary clarifications. We believe that their comments helped strengthen the credibility and robustness of our findings.

## Ethical approval

Not applicable.

## Consent

Not applicable.

## Source of funding

No external funding was received for this study.

## Author contribution

I.-W.C. and K.-C.H. wrote the main manuscript text. H.-T.C. and C.-L.K. prepared figure 1. All authors read and approved the final version of the manuscript.

## Conflicts of interest disclosure

The authors declare no conflicts of interest.

## Research registration unique identifying number (UIN)

Not applicable.

## Guarantor

Kuo-Chuan Hung.

## Data availability

The datasets used and/or analyzed in the current study are available from the corresponding author upon reasonable request.

## Provenance and peer review

Not applicable.
